# Genotype characteristics and immunological indicator evaluation of 311 hemophagocytic lymphohistiocytosis cases in China

**DOI:** 10.1186/s13023-020-01390-z

**Published:** 2020-05-06

**Authors:** Jia Zhang, Yuan Sun, Xiaodong Shi, Rui Zhang, Yini Wang, Juan Xiao, Jing Cao, Zhuo Gao, Jingshi Wang, Lin Wu, Wei Wei, Zhao Wang

**Affiliations:** 1 Department of Hematology, Beijing Friendship Hospital, Capital Medical University, 95 Yong An Road, Xicheng District, Beijing, 10050 China; 2 Department of Hematology, Beijing Jing Du Children’s Hospital, Beijing, China; 3 Department of Hematology, Capital Institute of Pediatrics, Beijing, China; 4 Hematology Oncology Center, Beijing Children’s Hospital, Capital Medical University, Beijing, China; 5 Clinical Epidemiology and Evidence-based Medical Center, Beijing Friendship Hospital, Capital Medical University, Beijing, China

**Keywords:** Primary hemophagocytic lymphohistiocytosis, Genetic testing, Rapid immunological indicators

## Abstract

**Background:**

Primary hemophagocytic lymphohistiocytosis (pHLH) is a genetic disorder that is classically diagnosed by genetic testing. Secondary HLH (sHLH) is usually caused by infections, malignancies, or autoimmune disorders, but may display some mutations or polymorphisms. Rapid immunological assays examining natural killer (NK) cell activity, degranulation function (CD107a), and protein expression related to genetic deficiencies have been recommended for early pHLH identification.

**Methods:**

A retrospective analysis of 311 HLH patients from a Chinese population was performed to evaluate the potential correlations between genetic testing and rapid immunological assays; genotyping characteristics, age of onset, and etiology were examined.

**Results:**

Among the 128 (128/311) patients who were positive in the genetic screening, the most frequently detected mutant gene was *UNC13D* (29%), followed by *LYST* (21%), *PRF1* (17%), and STXBP2 (10%). Among pHLH patients (*n* = 39), the majority (67%) had *PRF1* and *UNC13D* defects. FHL-2 was predominant (12/27, 44%) in patients aged under 18, while FHL-3 was the most common (6/12, 50%) in adults. Differences in genetic variant types and etiological components were noted in HLH patients based on the age of onset. NK cell activity and CD107a were observed to show a consistent trend (*P*_trend_ < 0.001) when grouping patients according to the severity of the genetic variant type. Moreover, NK cell activity was generally consistent within a certain range of ΔCD107a values (*P*_*trend*_ < 0.001). The PPV for bi-allelic degranulation gene mutations in patients with CD107a < 5% was 38.9% (7/18), while the PPV in patients with CD107a ≤10% was 16.7% (13/78). The PPV for pHLH was 41.4% (29/70) with NK cell activity ≤13%. To further evaluate the diagnostic efficacy of NK cell activity assay in pHLH, a receiver operating characteristic (ROC) curve was generated and showed an area under the curve (AUC) of 0.872, and the optimal cutoff value was determined to be 13.425% with a sensitivity of 84.21% and specificity of 80.67% when the corresponding Youden index was maximized. Flow cytometry screening for deficient proteins, including perforin, SAP, and XIAP, showed a relatively high sensitivity (83.33–93.33%). The positive predictive values (PPVs) of perforin and XIAP were relatively low (20.83–26.92%), but the negative predictive values (NPVs) for all three were excellent (all > 98%).

**Conclusions:**

Various immunological indicators have different clinical prediction and application values for the diagnosis of pHLH. The degree of reduction of immunological indicators also needs attention, and choosing appropriate cutoff value may be of important significance in guiding clinical judgment for pHLH.

## Background

Hemophagocytic lymphohistiocytosis (HLH) is a life-threatening hyperinflammatory syndrome caused by excessive macrophage and lymphocyte activation and can be attributed to various causes. The disorder is usually divided into two categories, primary/hereditary (pHLH), which is autosomal and/or X-linked recessive, and secondary/acquired (sHLH), which is attributed to underlying diseases (e.g., infections, malignancies, or autoimmune disorders). In pHLH, pathogenesis is mainly attributed to excessive immune system activation due to a reduction or absence of natural killer (NK) cell, and cytotoxic T lymphocytes (CTL) functions caused by genetic defects [1]. To diagnose pHLH, the Histiocyte Society recommends utilizing genetic testing, with at least 12 pHLH-related genes (including *PRF1*, *UNC13D*, *STX11*, *STXBP2*, *RAB27A*, *LYST*, *AP3B1*, *SH2D1A*, *BIRC4*, *ITK*, *CD27*, and *MAGT1*) recognized currently [2]. In addition, more primary immunodeficiency disorder (PID)-associated genes have been reported [3–5] with an increasingly in-depth understanding of PID-associated HLH. Furthermore, HLH can be considered a threshold disease, with genetic factors and other multiple endogenous and exogenous components interplaying until a critical point is reached, with sHLH also considered to have a certain degree of genetic underpinning [6].

For the early identification and prediction of pHLH, various rapid immunological assays, such as NK cell activity assay, CD107a degranulation assay, and screening for deficiency in protein expression of relevant genes, have been recommended due to their fast turnaround time. Among them, a reduction or absence of NK cell activity is considered an important HLH indicator that reflects immunodeficiency. To assess NK cell activity, various approaches, such as Cr^51^ radioimmunoassay [7–9], lactate dehydrogenase (LDH) release assay [10, 11], and various types of flow cytometry [12–17] have been adopted under different conditions and parameters, with different degrees of sensitivity and specificity. Thus far, no uniform measurement standard has been adopted. CD107a, also called lysosomal-associated membrane protein-1 (LAMP-1), is a major component of vascular membrane proteins. Utilizing flow cytometry analysis to quantify degranulation in cytotoxic cells can help rapidly and clearly distinguish conditions with granulocytosis dysfunction [e.g., familial hemophagocytic lymphohistiocytosis types 3–5 (FHL-3–5), Griscelli syndrome type 2 (GS-2), Chediak–Higashi syndrome (CHS), or Hermansky-Pudlak syndrome type 2 (HPS2)] from conditions without granulocytosis dysfunction [e.g., familial hemophagocytic lymphohistiocytosis type 2 (FHL-2), X-linked lymphoproliferative disease (XLP) with deficiency in SLAM associated protein (SAP) or X-linked inhibitor of apoptosis protein (XIAP), or sHLH] [18]. Thus, the CD107a assay is being considered for inclusion in the diagnostic criteria for HLH. In addition, screening for perforin, SAP, XIAP, Munc13–4, synthaxin-11, and Munc18–2 expression via flow cytometry or western blot can also provide rapid identification of relevant defective genes. Once immunological indicators suggest the presence of a genetic basis for HLH, subsequent genetic identification should be performed. The two approaches need to be combined and mutually validated to guide the treatment strategies.

This study analyzed the results of genetic testing and rapid immunological assays in 311 patients with HLH in China to evaluate the accuracy and diagnostic efficacy of these immunological assays and to explore the characteristics of genotyping, age of onset, and etiology.

## Materials and methods

### Samples

HLH patient genetic and immunological testing data (*n* = 311) that were obtained from patients treated at the Beijing Friendship Hospital, Capital Medical University (Beijing, China), the Beijing Jingdu Children’s Hospital (Beijing, China), the Affiliated Children’s Hospital of Capital Institute of Pediatrics (Beijing, China), and the Beijing Children’s Hospital, Capital Medical University (Beijing, China) from April 2015 to February 2018 were retrospectively analyzed. All patients met the HLH-2004 diagnostic criteria recommended by the Histiocyte Society [19]. Correlations and accuracy between genetic testing results and rapid immunological indicators, including NK cell activity, CD107a, and protein expression (perforin, SAP, and XIAP), were examined. Some patients had multiple results for a given immunological assay, but only the first valid result was selected.

### Genetic testing and variant analysis

High-throughput sequencing (targeted gene sequencing panels and WES) and Sanger sequencing were utilized to evaluate the 311 HLH patients. Mutations associated with pHLH, including *PRF1*, *UNC13D*, *STX11*, *STXBP2*, *LYST*, *RAB27A*, *ADTB3A*, *SH2D1A*, *BIRC4*, *ITK*, *CD27*, and *MAGT1*, were examined and interpreted using the Human Gene Mutation Database (HGMD; http://www.hgmd.cf.ac.uk/), the Single Nucleotide Polymorphism database (dbSNP; http://www.ncbi.nlm.nih.gov/snp/), and Ensemble (http://asia.ensembl.org/index.html). The 1000 Genomes Project, Exome Aggregation Consortium (ExAC), and NHLBI Exome Sequencing Project (ESP6500) were referenced for variant frequencies; while Sorting Intolerant From Tolerant (SIFT) and PolyPhen-2 were used for SNP predictions. Variant pathogenicity classifications were determined in accordance with the standards and guidelines recommended by the American College of Medical Genetics and Genomics (ACMG) [20]. The genetic variants selected in this study were those identified by pathogenicity analysis as pathogenic, likely pathogenic, or uncertain significance. These variants also met at least one of the following requirements: (1) the mutation in the population has a minor allele frequency ≤ 0.01 based on the annotation from the ALlele FREquency Database (ALFRED); (2) the mutation was determined to be harmful/possibly harmful by at least one of the two prediction algorithms (SIFT or Polyphen-2); (3) the mutation had been reported to be pathogenic in the literature; or (4) the mutation was a nonsynonymous mutation that had not been previously reported in the literature and had no allele frequency annotation or pathogenicity prediction.

### Etiological analysis

Differences in etiological components of 311 HLH patients, such as pHLH, infection, malignancy, autoimmune disorders, and other unknown causes, in different age groups, including ≤2 years old, > 2 to < 18 years old, and ≥ 18 years old, were analyzed. Additionally, gene mutations in various underlying diseases associated with sHLH were also examined.

### Rapid immunologic assays

#### NK cytotoxicity assay

To detect NK cytotoxicity, a new flow cytometry assay (China National Invention Patent, No. ZL201410005008.7) was used [21]. For this assay, K562 cells, as the standard target for NK cells, were engineered to stably express enhanced green fluorescent protein (EGFP) via lentiviral transfection. Then the EGFP-K562 cells (target cells) were co-incubated with the peripheral blood mononuclear cells (PBMCs, effector cells) of HLH patients based on the specific effector: target cell ratio. After the co-culture of the target and effector cells, the percentage of apoptotic EGFP-K562 cells reflected NK cytotoxicity. To determine the proportion of apoptotic target cells, samples were labeled with Annexin V-PE, and 7-amino-actinomycin D (7-AAD) (eBioscience, San Diego, CA, USA) and flow cytometry were performed.

#### NK cell degranulation assay

PBMCs were isolated and co-incubated with a specific ratio of K562 cells for stimulation or incubated with medium alone (control group). Samples were then labeled with anti-CD3-fluorescein isothiocyanate (FITC), anti-CD8-allophycocyanin (APC), anti-CD56-PC5.5, and anti-CD107a-PE (eBioscience) for flow cytometry. For analysis, CD3^−^CD56^+^ NK cells were gated and evaluated by determining the amplitude change of surface expression of CD107a (NK-ΔCD107a), with and without K562 stimulation. The reference intervals were defined as < 5% being deficient, ≥ 5% and ≤ 10% being abnormal, and > 10% being normal [18].

#### Perforin, SAP, and XIAP expression in NK cells

PBMCs were isolated and labeled with anti-CD3-FITC, anti-CD8-PerCP, and anti-CD56-APC before cell fixation and cell membrane rupture. The intracellular proteins were further labeled with anti-perforin, anti-SAP, and anti-XIAP. Additionally, these proteins were quantified in NK cells using flow cytometry.

### Statistical analysis

Analyses were performed using the IBM SPSS Statistics 19.0 software (IBM SPSS Inc., Chicago, IL). Independent-samples *t*-test was used for quantitative data with a normal distribution. Differences in etiology were analyzed using a non-parametric Chi-square test. A multi-sample distribution trend comparison of non-normally distributed data was performed using a Jonckheere-Terpstra test. Receiver operating characteristic (ROC) curves were generated and used to determine the optimal threshold (cutoff values) of NK cell cytotoxicity that would identify pHLH patients with maximum sensitivity and specificity (Youden index). For the flow cytometry analyses, the sensitivity, specificity, positive predictive values (PPVs), and negative predictive values (NPVs) were determined for predicting genetic mutations by perforin, SAP, or XIAP expression based on the laboratory-generated normal ranges for each test. Statistical significance is defined as *P* < 0.05, and the significance threshold was corrected for pairwise comparisons between multiple groups.

## Results

### General information

**Fig. 1:**
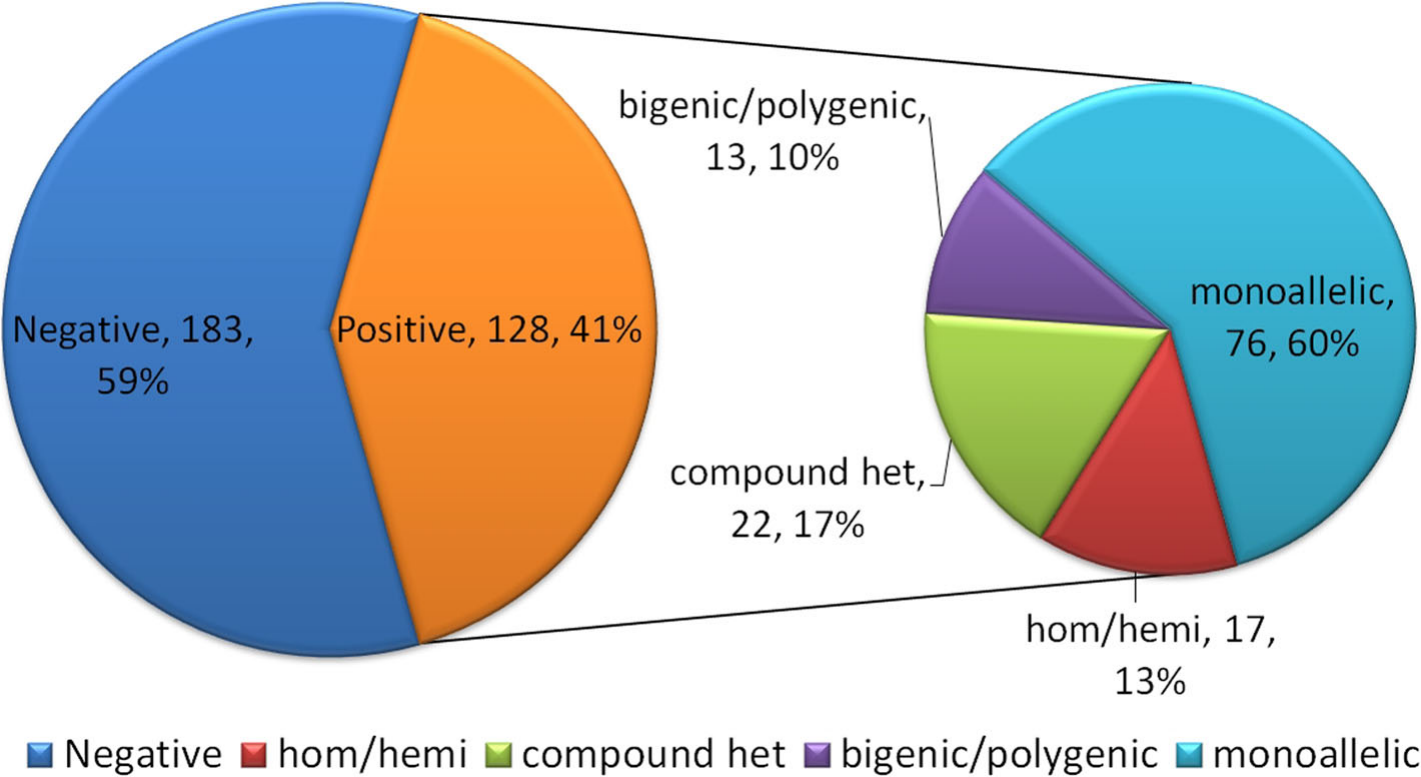
Pie charts showing the proportions of patients with negative/positive genetic findings (left) and the different types of gene mutations among those with positive genetic findings (right). Hom: homozygous; hemi: hemizygous; and het: heterozygous

The examined HLH patients (*n* = 311) had a male-to-female ratio of 167:144, with an age range from 2 months to 74 years old and a median age of 19 years. Genetic testing was performed on all of the patients, with 128 patients, 67 males and 61 females, found to have potential disease-related variants (Supplementary Table S1), with an age range of 2 months to 70 years and a median age of 14.5 years (≤ 2 years of age, *n* = 26; > 2 to < 18 years old, *n* = 46; ≥ 18 years of age, *n* = 56). The type of variants included frameshift/non-frameshift, nonsense, missense, and splice-site (upstream and downstream of 5′ splice sites) variants. Furthermore, 183 cases lacked genetic findings (Fig. 1).

### Forms of genetic variants and their distributions in different age groups

**Fig. 2:**
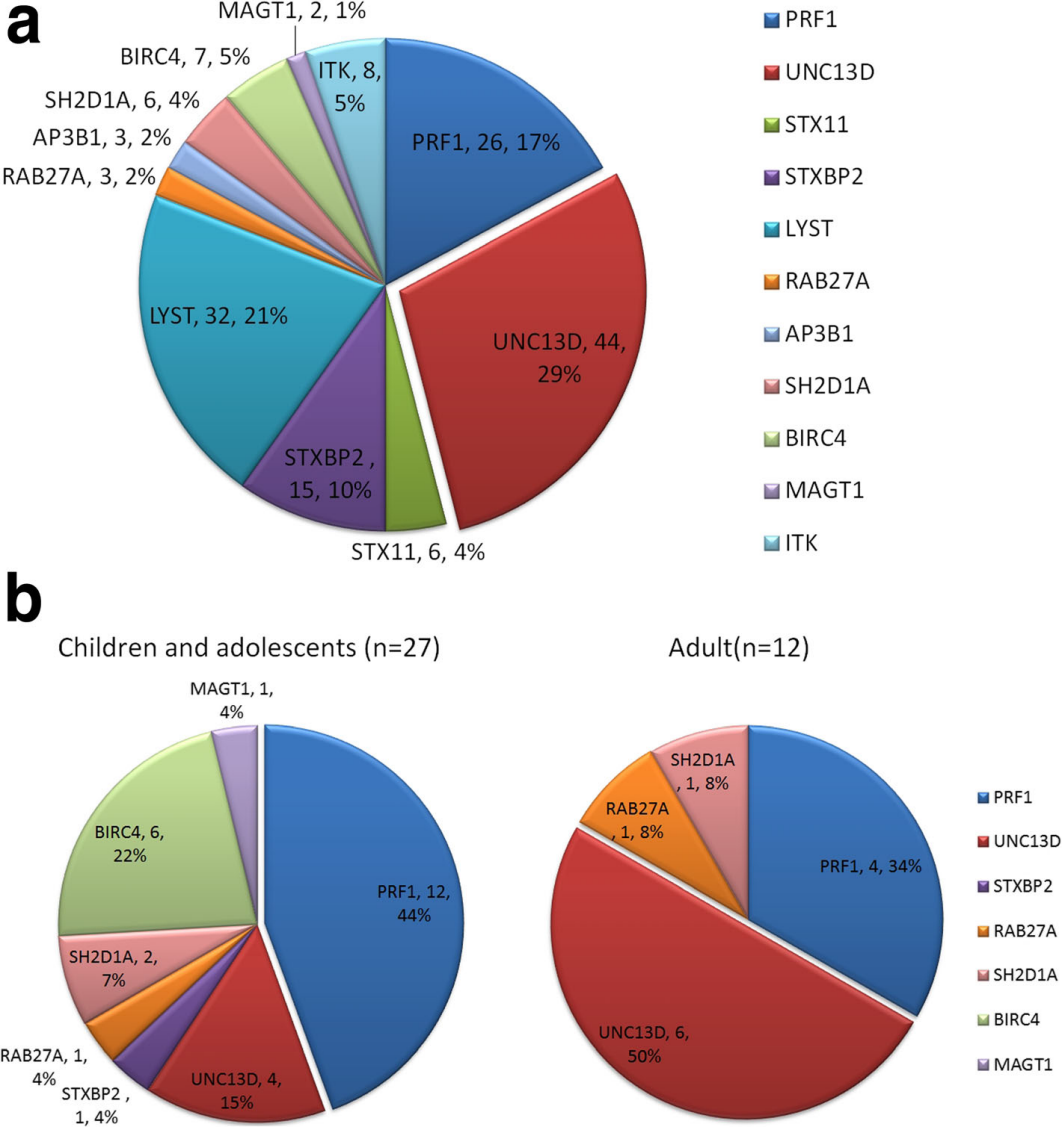
Pie charts showing the frequencies of the gene mutations. **a** Frequencies of the different gene mutations associated with HLH. **b** Frequencies of the pathogenic genes in pHLH patients under 18 (children and adolescents) (left) and adult patients (right)

Among the 128 cases with pathogenic or contributing mutations, 17 cases (13%) had homozygous/hemizygous mutations; 22 cases (17%) had compound heterozygous mutations; 13 cases (10%) had bigenic/polygenic heterozygous mutations, and 76 cases (60%) had heterozygous monogenic mutations (Fig. 1). The most frequently detected mutant gene was *UNC13D* (29%), followed by *LYST* (21%), *PRF1* (17%), and *STXBP2* (10%; Fig. 2a). For the 39 pHLH cases, the main pathogenic gene was *PRF1* (16/39, 41%), followed by *UNC13D* (10/39, 26%) and *BIRC4* (6/39, 15%). In addition, there were differences between children and adolescents (< 18 years old) and adults (≥18 years old) in pHLH-related genes, with PRF1 (12/27, 44%) being prominent in children and adolescents, followed by BIRC4 (6/27, 22%) and UNC13D (4/27, 15%); while in adults, UNC13D (6/12, 50%) was the most prevalent, followed by PRF1 (4/12, 33%; Fig. 2b). When examining the distributions of different forms of genetic mutations in different age groups, homozygous/hemizygous mutations and compound heterozygous mutations (diagnosed with pHLH) were found to occur predominantly in early-onset (27/39, 69%), with an onset age of 13.11 ± 12.91 years. This onset age was significantly younger than the onset age associated with other types of gene mutations (19.99 ± 16.86 years; *P* = 0.025). Furthermore, in pHLH patients with large-fragment variants, the onset age was as young as 8.84 ± 8.29 years, which was significantly younger than in other pHLH patients (*P* = 0.033).

### Stratification of etiology and genetic background

**Fig. 3:**
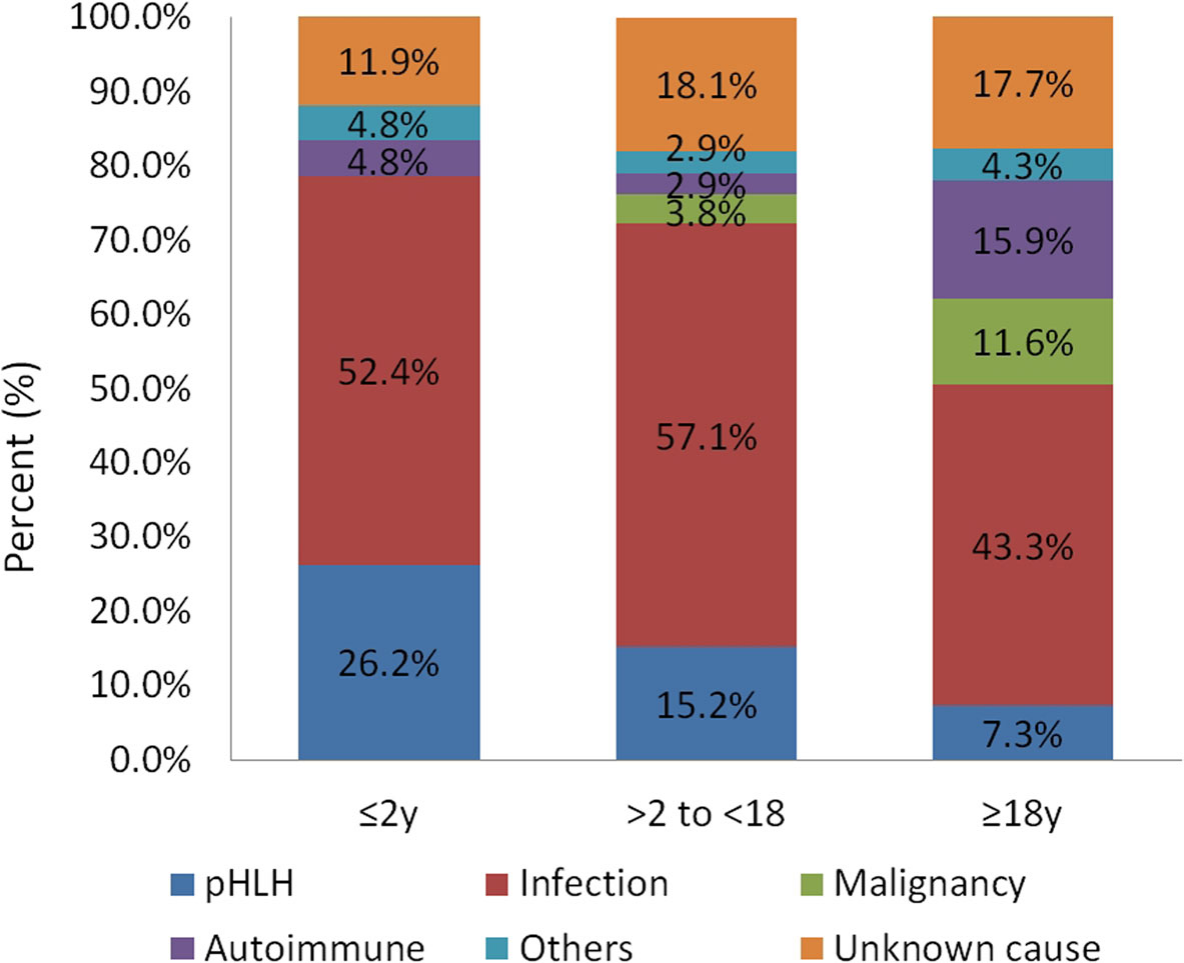
Comparison of the etiology of HLH at different onset ages

**Fig. 4:**
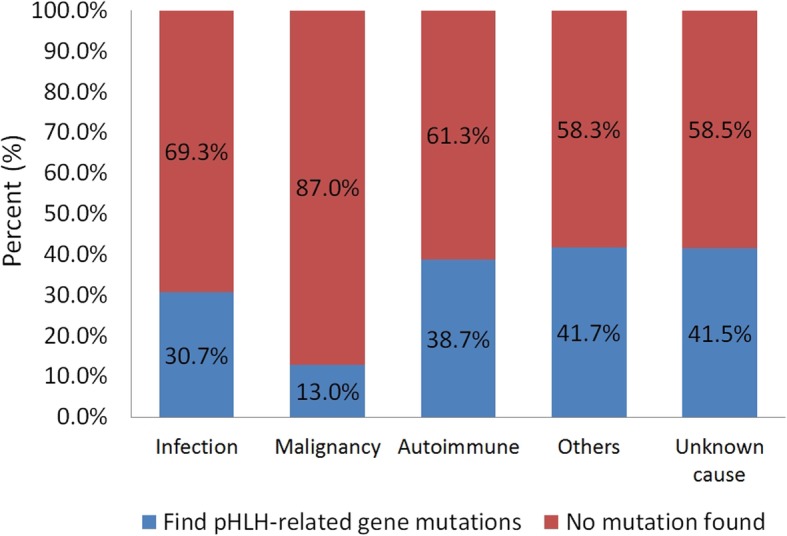
Gene abnormalities in different underlying diseases associated with secondary HLH

In younger age groups, there was a significantly higher proportion of pHLH patients, including 26.2% of those ≤2 years (11/42) and 15.2% of those > 2 to < 18 years (16/105), when compared to the ≥18 years age group (12/164, 7.3%; *P* = 0.003). Furthermore, while the proportion of pHLH in ≤2 year group appeared to be higher than that of the > 2 to < 18 year group, no significant difference was found between the two groups (*P* = 0.096). In addition, the proportion of pHLH cases among HLH patients under one-year-old (*n* = 8) and over 40 years old were found to be 75% (6/8) and 3.1% (1/32), respectively. Moreover, when examining infection associated HLH, no significant difference was noted between the different age groups: ≤ 2 years (22/42, 52.4%) vs. > 2 to < 18 years (60/105, 57.1%; *P* = 0.713); ≤ 2 years (22/42, 52.4%) vs. ≥ 18 years (71/164, 43.3%; *P* = 0.303); > 2 to < 18 years (60/105, 57.1%) vs. ≥ 18 years (71/164, 43.3%; *P* = 0.033, with *P* < 0.017 being the adjusted value for statistical significance). However, the proportion of infection related HLH in children and adolescents (< 18 years, 55.8%) is higher than that in adults (43.3%, *P* = 0.031). Within the three groups of infection associated HLH, more than 90% of the patients had Epstein-Barr virus (EBV) associated HLH. Additionally, the proportion of malignancy associated HLH gradually increased with age, with no cases of malignancy associated HLH found in the ≤2 years age group. In the ≥18 years age group, the proportion of malignancy associated HLH (19/164, 11.6%) was significantly higher than in the > 2 to < 18 years group (4/105, 3.8%; *P* = 0.019). Moreover, the proportion of autoimmune disease associated HLH in the adults (26/164, 15.9%) was significantly higher than in patients > 2 to < 18 years (3/105, 2.9%; *P* < 0.001), but had no significant difference compared with patients ≤2 years (2/42, 4.8%; *P* = 0.077; Fig. 3). Furthermore, a certain degree of genetic contribution was found in sHLH patients with all types of underlying diseases, including infection (47/153, 30.7%); malignancy (3/23, 13.0%); autoimmune disease (12/31, 38.7%); others (5/12, 41.7%), such as Langerhans cell histiocytosis, PID, pregnancy, and drug-associated; and unknown reason (22/53, 41.5%; Fig. 4).

### Differences in NK cell activity among different mutation types

**Fig. 5:**
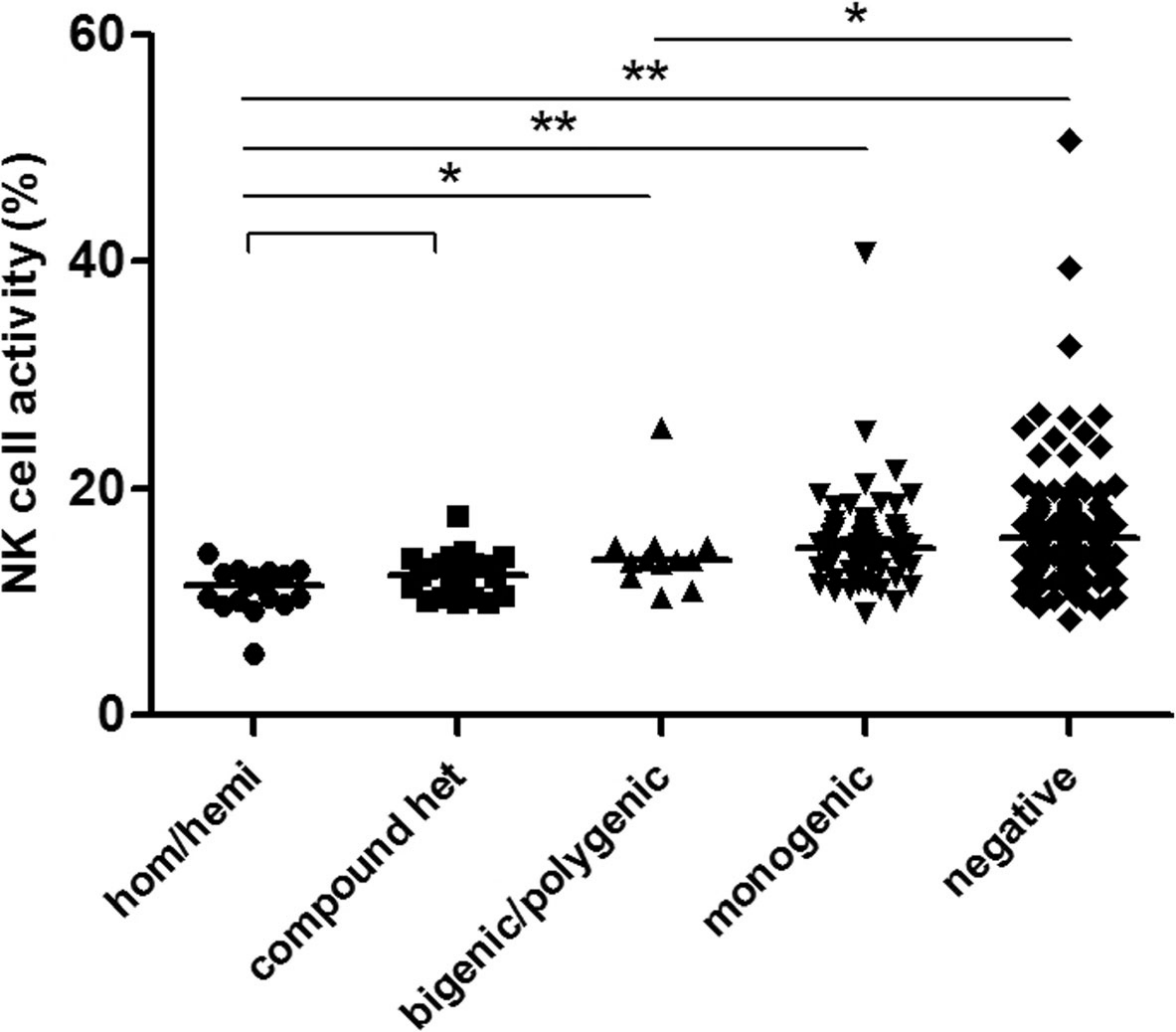
Comparison of NK cell activities in patients with different types of mutations. Error bars in the scatter plot indicate the median for different groups. A Jonckheere-Terpstra test was used for statistical analysis. *P < 0.05, ** *P* < 0.01

Of the 311 patients, 307 had their NK cell activity evaluated. When grouping these patients based on the severity of the genetic mutation type, the NK cytotoxicity showed a consistent trend as the mutation type became less severe (*P*_trend_ < 0.001). When performing a pairwise comparison of different groups, patients with homozygous/hemizygous mutations and compound heterozygous mutations (*n* = 38) had a lower NK cell activity relative to patients with bigenic/polygenic heterozygous mutations (*n* = 12; *P* = 0.014), heterozygous monogenic mutations (*n* = 75; *P* < 0.001), or those who had negative findings during genetic screening (*n* = 182; *P* < 0.001). Moreover, in patients with bigenic/polygenic heterozygous mutations, NK cell activity was lower than in patients that tested negative for mutations (*P* = 0.023). However, when comparing NK cell activity between patients with heterozygous monogenic mutations and bigenic/polygenic heterozygous mutations (*P* = 0.275) or patients that tested negative for mutations (*P* = 0.132; Fig. 5), no significant difference was noted.

### Relationship between CD107a and variants of degranulation-related genes

**Fig. 6:**
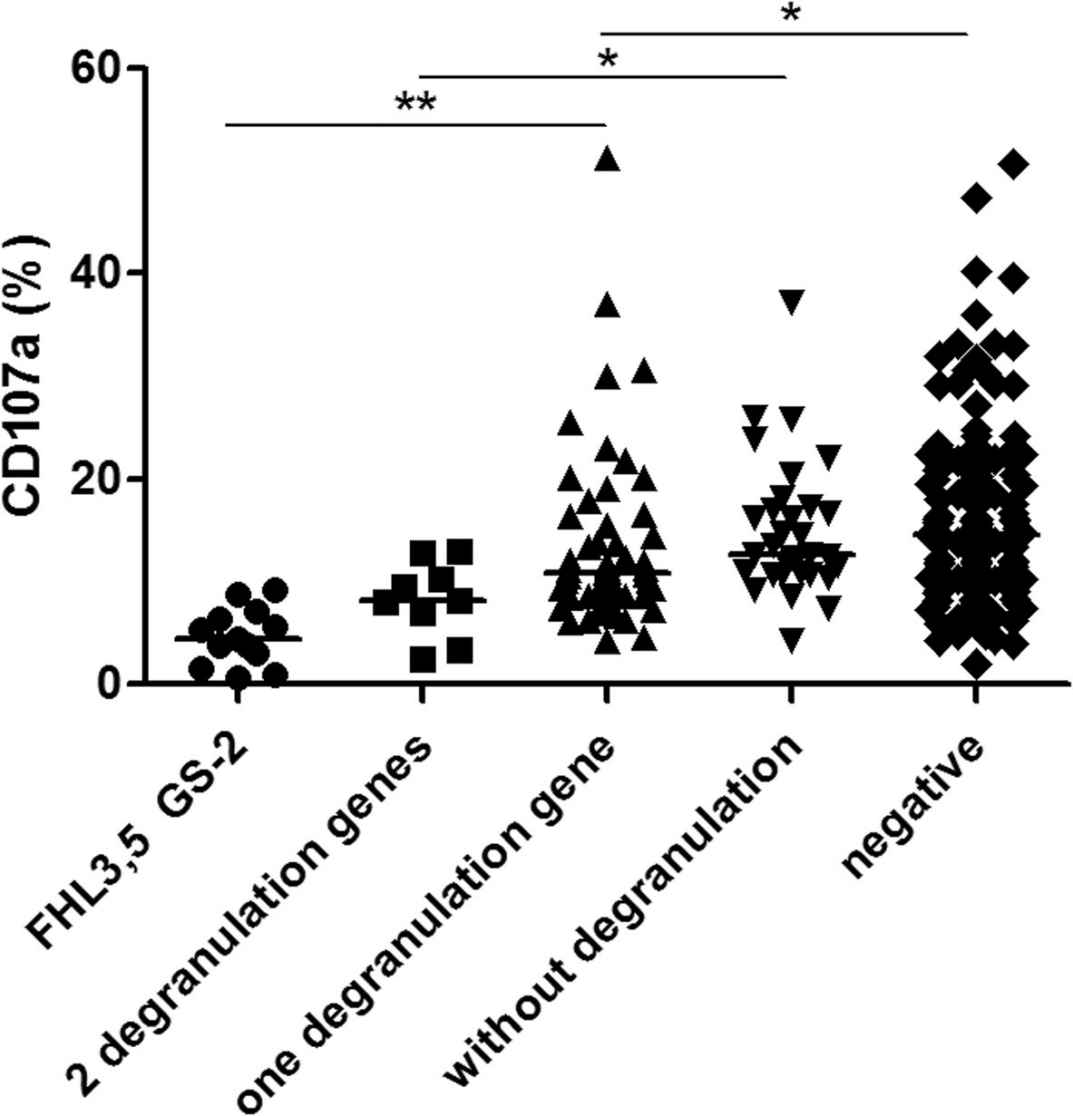
Comparison of CD107a in patients with different types of mutations in degranulation-related gene

A total of 260 patients completed the degranulation function test. After grouping patients according to the severity of the genetic variant type in degranulation-related genes, it was found that the NK-ΔCD107a results also showed a consistent trend (*P*_trend_ < 0.001). Additionally, pairwise comparison of the NK-ΔCD107a results was assessed and significant differences were identified between biallelic variants in degranulation-related genes (FHL-3,5; GS-2, *n* = 13) and heterozygous monogenic variants involving one degranulation-related gene (*n* = 55, *P* < 0.001), between “potential digenic mode”–heterozygous variants involving two degranulation-related genes (*n* = 9) and variants not involving degranulation-related genes (*n* = 32, *P* = 0.011), and between heterozygous monogenic variants involving one degranulation-related gene and negative findings following genetic screening (*n* = 151, *P* = 0.028). However, no significant differences were noted when comparing biallelic variants in degranulation-related genes vs. digenic variants in degranulation-related genes (*P* = 0.150); digenic variants in degranulation-related genes vs. heterozygous monogenic variants involving one degranulation-related gene (*P* = 0.218); or heterozygous monogenic variants involving one degranulation-related gene vs. variants not involving degranulation-related genes (*P* = 0.127). Moreover, there were no significant differences between patients who had variants in genes not related to degranulation and patients that tested negative for variants (*P* = 1.000; Fig. 6).

### Consistency between NK cell activity and CD107a

Of the 311 examined patients, 256 patients had both NK cell activity and CD107a detection assays performed. After eliminating the data of patients with *PRF1* mutations, the remaining 232 patients were analyzed. Based on the defined ΔCD107a intervals (< 5%, deficient; ≥ 5% and ≤ 10%, abnormal; > 10%, normal), the NK cell activity results were divided into three groups, with an increasing trend noted (*P*_trend_ < 0.001). A pairwise comparison between groups showed that the normal (≤ 10%) group was significantly different from the deficient (< 5%; *P* < 0.001) and abnormal (≥ 5% and ≤ 10%; *P* < 0.001) groups. Additionally, the NK cell activity of the deficient (< 5%) group appeared to be lower than the abnormal (≥ 5% and ≤ 10%) group, but no significant difference was noted (*P* = 0.068) (Table 1).

**Table 1:** Comparison of NK cells activity among stratified CD107a expression

ΔCD107a expression (%)	< 5	≥ 5 and ≤ 10	> 10
*n*	17	58	157
Median of NK cell activity (%)	12.77	13.84	15.88
Range of NK cell activity (%)	5.40–16.13	9.51–26.43	9.00–50.75
*P* value of NK cell activity	*0.068*^*a*^	< 0.001**^b^	< 0.001**^c^

*n*, number. Jonckheere-Terpstra test was implemented. ** *P* < 0.01

^a^ΔCD107a < 5% group vs. the ≥5% and ≤ 10% group

^b^ΔCD107a ≥ 5% and ≤ 10% vs. the ≥10% group

^c^ΔCD107a < 5% vs. the > 10% group

### Diagnostic efficacy of CD107a and the NK cell activity assay

**Fig. 7:**
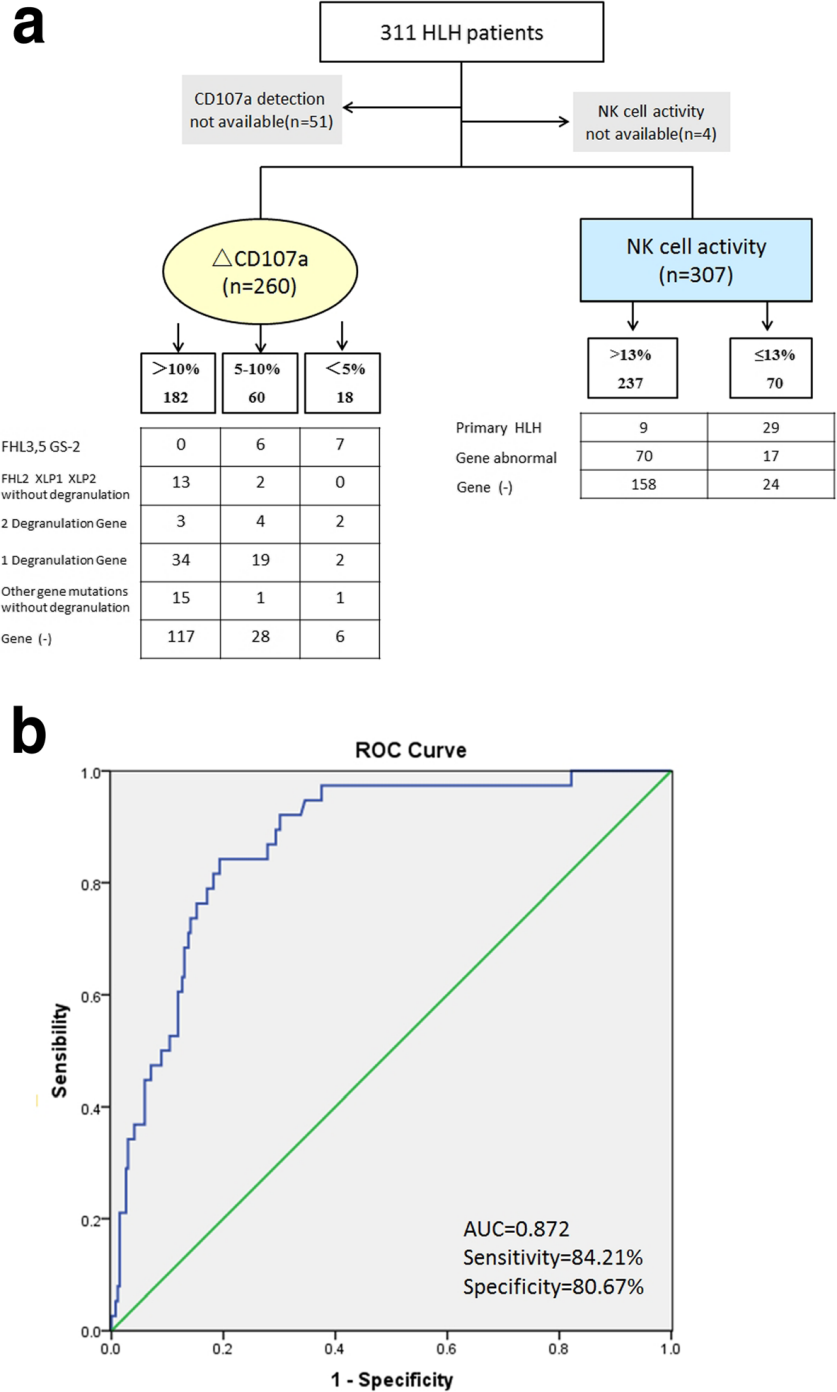
Determination of diagnostic efficacies of CD107a and NK cell activity assays for the presence of genetic abnormalities in patients with HLH. **a** Comparison of CD107a and NK cell activity assays with genetic testing results. **b** Empirical ROC curves for the detection of NK cell activity

In patients with confirmed FHL-3,5 or GS-2 (*n* = 13), a ΔCD107a > 10% results was not identified. Furthermore, in confirmed FHL-2 and XLP patients without degranulation-related gene mutations (*n* = 15), no ΔCD107a < 5% result was noted, and 13 out of 15 patients (86.7%) had a ΔCD107a > 10% result. Among the 9 patients whose mutations involving two degranulation-related genes, six (6/9, 66.7%) were ΔCD107a abnormal or deficient (5–10% or < 5%, respectively). Additionally, most of the 55 patients with one degranulation-related gene mutation showed a ΔCD107a > 10% result (34/55, 61.8%), followed by a ΔCD107a 5–10% result (19/55, 34.5%), and only 2 patients had a ΔCD107a < 5% result (2/55, 3.6%). For the patients without a degranulation-related gene mutation (*n* = 17) and for the patients that tested negative (*n* = 151), the majority showed a ΔCD107a > 10% results, accounting for 15/17 (88.2%) and 117/151 (77.5%), respectively. To evaluate the diagnostic efficacy of NK cell activity testing in pHLH, a ROC curve was constructed. The area under the curve (AUC) was 0.872 (*P* < 0.001) and the maximum Youden index was 0.649, with a corresponding sensitivity and specificity of 84.21 and 80.67%, respectively, and an optimal NK cell activity cutoff value of 13.425% (Fig. 7a and b).

### Accuracy evaluation between genetic testing and perforin, SAP, and XIAP protein screening

**Fig. 8:**
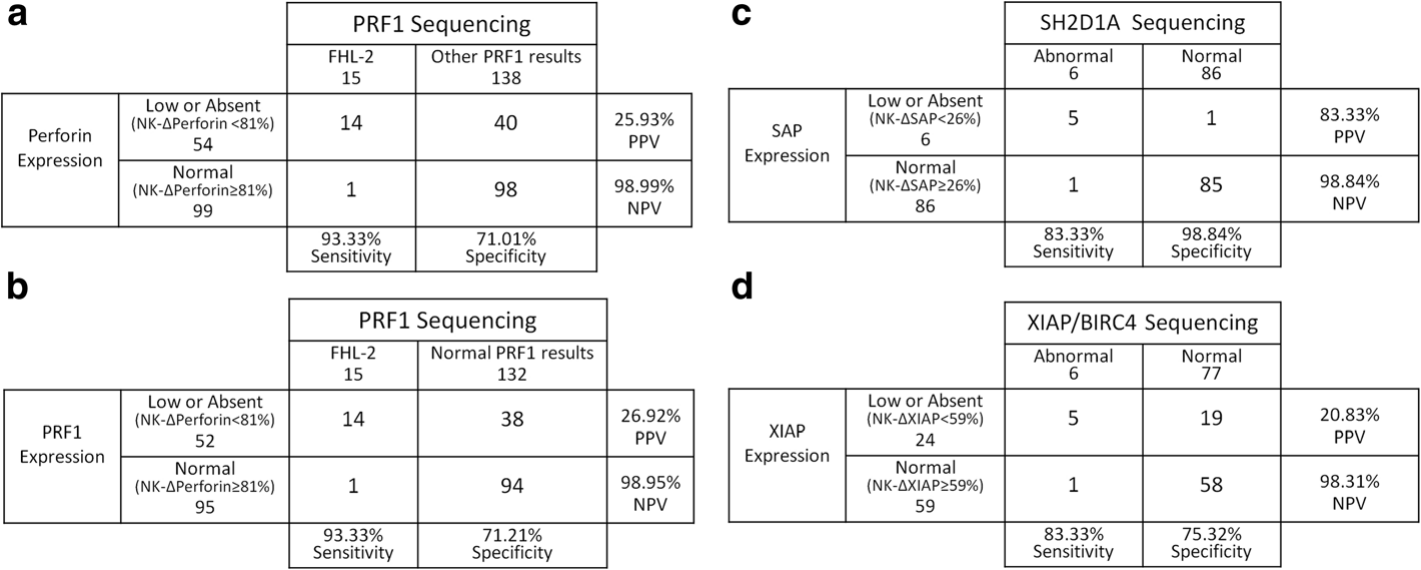
Diagnostic accuracy of perforin/SAP/XIAP expression for the presence of genetic abnormalities in patients with HLH. Determination of sensitivity, specificity, PPV, and NPV for low or absent (**a**) perforin expression to distinguish FHL-2 from all other PRF1 sequencing results; (**b**) perforin expression to distinguish FHL-2 from normal PRF1 sequencing results; (**c**) SAP expression to distinguish patients with abnormal SH2D1A sequencing results from normal ones; and (**d**) XIAP expression to distinguish patients with abnormal XIAP/BIRC4 sequencing results from normal ones. PPV, positive predictive value; NPV, negative predictive value

Some patients completed protein expression testing for perforin (*n* = 153), SAP (*n* = 92), and XIAP (*n* = 83). The diagnostic accuracy (sensitivity, specificity, PPV, and NPV) was evaluated based on the laboratory-generated normal ranges for each indicator. Perforin, SAP, and XIAP had a relatively high sensitivity (83.33–93.33%). Furthermore, the PPVs for perforin and XIAP were lower (26.92 and 20.83%) than that of SAP (83.33%), but all three had excellent NPVs (all > 98%) (Fig. 8).

## Discussion

Of the Chinese HLH patients who underwent genetic testing (*n* = 311), 128 cases (41%) had disease-associated mutations (Fig. 1; Supplementary Table S1), of which 39 were pHLH patients, with FHL-2 (16/39, 41%) and FHL-3 (10/39, 26%) being the most common. In a study examining a Japanese population, FHL-2 and FHL-3 were reported to account for 55 and 32% of FHL patients, respectively [22]. Additionally, in a study examining a Korean population, *UNC13D* (FHL-3) was shown to be a major pathogenic gene in FHL [23]. In the present study, we found that FHL-2 (12/27, 44%) was predominant in children and adolescents (< 18 years old), while FHL-3 (6/12, 50%) was the most common in adults (≥18 years old) (Fig. 2b). The onset age of FHL-2 patients is earlier than that of other types of FHL, mainly due to the *PRF1* mutation causing more severe cytotoxicity damage. Furthermore, we found that hemizygous *BIRC4* mutations (XLP-2) also account for a considerable proportion (6/27, 22%) of pHLH patients less than 18 years old. It had been reported that the number and function of T cells and NK cells were decreased and impaired in varying degrees in XLP-2 patients, and the symptoms such as recurrent infection and HLH associated with chronic EBV disease occurred at an early age [24–26]. Moreover, in the present study, mutation distribution was also examined for different age groups. Among them, nearly 70% of pHLH (27/39, 69%) occurred before the age of 18. Furthermore, pHLH patients with large-fragment variants had an earlier onset age than did pHLH patients without large-fragment variants (*P* = 0.033).

To explore HLH etiology, different age groups (≤ 2 years, > 2 to < 18 years, and ≥ 18 years; Fig. 3) were examined, and pHLH was found to mainly occur at a young age, with 26.2% (11/42) of HLH cases being pHLH in the ≤2 year group. Morimoto et al. [27] showed that the proportion of FHL in pediatric HLH patients under 1 year of age is nearly 50% in Japan. Herein, the proportion of pHLH cases among HLH patients under one-year-old (*n* = 8) was as high as 75% (6/8), thus suggesting that in HLH patients under 1 year, pHLH should be highly suspected. Moreover, the proportion of pHLH was roughly inversely correlated with age, with 15.2% of > 2 to < 18 -year group (16/105) and 7.3% of ≥18 years age group (12/164). Notably, among adult HLH patients over 40 years old, the proportion of pHLH is very low, only 3.1% (1/32).

HLH can also be associated with various diseases. In the present study, infection related HLH group was diagnosed after excluding other causes such as pHLH, malignancy, and autoimmune disease. Infection was a major cause of HLH in the three different age groups with no significant difference noted between groups. However, the proportion in younger age groups (< 18 years) is higher than that in adults (*P* = 0.031). Infection associated HLH is mostly attributable to EBV infection, which is highly prevalent among Asian populations [28, 29] and accounted for more than 90% of the infection associated HLH cases identified herein. Additionally, the proportion of malignancy associated HLH gradually increased with age. In a large-scale Japanese study examining incidence rates of lymphoma associated HLH in different age groups, lymphoma associated HLH was determined in 68% of individuals > 60 years, 38% of individuals 30–59 years, 10% of individuals 15–29 years, and 0% of patients under 14 years [30]. In our study, malignancy associated HLH accounted for 0% of ≤2 years age group (0/42), 3.8% of > 2 to < 18-year group (4/105), and 11.6% of ≥18 years age group (19/164), respectively.

Moreover, this study showed a certain proportion of pHLH-related gene mutations in sHLH patients with different types of underlying diseases (Fig. 4). Recently, HLH has been proposed to be a threshold disease, with there being no clear boundary between pHLH and sHLH because both essentially have the same outcome and are caused by a combination of multiple underlying diseases and pathogenic mechanisms [6]. Notably, this study proposed that sHLH also has a certain genetic background, such as heterozygous variants or polymorphisms in pHLH-related genes, and that sHLH is induced after a “second hit” from an exogenous trigger (e.g., a viral infection).

To compare the correlation between gene mutations and cellular function indicators (i.e., NK cell activity and CD107a), the patients were grouped based on the severity of the genetic mutation type in pHLH-related genes and in degranulation-related genes. In general, cytotoxicity levels were increased as the severity decreased (*P*_trend_ < 0.001; Figs. 5 and 6). The trend of CD107a, to a certain extent, support the findings presented by Zhang et al. [31] that showed that degranulation-related bigenic heterozygous mutations cause synergistic damage, and are attributed to digenic inheritance. To assess the consistency between the NK cell activity assay and CD107a results, samples were regrouped based on the defined ΔCD107a intervals (< 5%, deficient; ≥ 5% and ≤ 10%, abnormal; and > 10%, normal) following the removal of patients with a *PRF1* mutation. While the NK cell activity was found to increase in a fashion that was consistent with the CD107a results (*P*_trend_ < 0.001) and both indicators reflect cytotoxic function, these two assays measure different things and are not mutually replaceable. NK cell activity reflects the direct killing ability of cytotoxic cells. Impaired or defective cytotoxicity caused by various pathways can lead to a decrease in NK cell activity. Moreover, this factor is also an important indicator of the HLH-2004 diagnostic criteria. Furthermore, CD107a reflects the exocytosis of cytolytic granules and is mainly a parameter for the rapid identification of defects in genes involved in the regulation of vesicle-mediated transport.

In terms of the diagnostic efficacy of the two assays (Fig. 7a and b), in the present study, a ΔCD107a > 10% was not observed in patients with confirmed FHL-3,5 and GS-2; while patients with confirmed FHL-2 and XLP, but without mutations in degranulation-related genes, did not have a ΔCD107a < 5%. Additionally, only 5.9% (1/17) of patients without degranulation-related gene mutations and 4.0% (6/151) of patients that tested negative for mutations had a ΔCD107a < 5%, thus suggesting that CD107a had significant values in exclusive diagnosis. To evaluate the diagnostic efficacy of NK cell activity assays in pHLH, the detection findings of 307 patients were compared with the genetic testing results, with a ROC curve generated with an AUC = 0.872 (*P* < 0.001) and a maximum Youden index of 0.649. The sensitivity and specificity were determined to be 84.21 and 80.67%, respectively, and the optimal NK-cell activity cutoff value was 13.425%. While Cr^51^ has been considered the gold standard for detecting NK-cell activity, it is difficult to be used universally because of its radioactive contamination and high cost. The new flow cytometry method used in the present study provides a rapid, user-friendly way for measuring NK cell cytotoxicity and has potential to serve as another testing approach in HLH patients [21]. Furthermore, it should be of note that the degree of reduction of cytotoxic function indicators also needs attention. Indeed, in the present study, the PPV for bi-allelic degranulation gene mutations in patients with CD107a < 5% was 38.9% (7/18), the PPV for bi-allelic degranulation gene mutations in patients with CD107a ≤10% was 16.7% (13/78), and the PPV for primary HLH was 41.4% (29/70) in patients with NK cell activity ≤13%. In clinical practice, in the presence of patients with severely decreased NK cell killing activity and/or defective degranulation function, HLH-related genes should be investigated first.

When examining flow cytometry for the screening of gene-related protein deficiencies (perforin, SAP, and XIAP expression; Fig. 8) in conjunction with associated genetic testing, a relatively high sensitivity (83.33–93.33%) and limited false-negative outcome were obtained. However, for the perforin and XIAP deficiency screenings, the PPVs were relatively low (20.83–26.92%). Therefore, a large number of patients with a positive primary screening would be included as suspected subjects for further genetic testing validation. Moreover, SAP deficiency screening showed a relatively high specificity (98.84%) and PPV (83.33%), thus suggesting that more than 80% of patients with low SAP expression may have an *SH2D1A* mutation. Importantly, the NPVs for perforin, SAP, and XIAP following deficiency screening were excellent (all > 98%), thus suggesting that > 98% of patients with a negative screening result can definitively be ruled out. Additionally, these protein deficiency screenings were also compared with previous findings from Rubin et al. [32] and Gifford et al. [33], some variability among centers and patients might account for variable PPVs. In addition, the degree of reduction of protein expression in the different mutation types of patients is different. Gifford et al. [33] also optimized the cutoff value of SAP and XIAP expression. Therefore, in consideration of sensitivity and specificity, choosing the appropriate cutoff value may be of great significance in guiding clinical judgment for pHLH. Of note, the protein deficiency screenings measure the quantity of expressed proteins to reveal target gene abnormalities. While most genetic defects cause a reduction in the quantity of the expressed proteins, some missense mutations may result in protein variants synthesized in a normal quantity [33]. Therefore, protein deficiency screenings have certain limitations, and genetic testing should still be performed in some patients with a typical XLP phenotype, but with normal protein expression and no other genetic evidence [33]. In view of these findings, some optimized immunological assays are worth examining as additional testing platforms. For example, researchers have proposed the use of a functional NOD2 signaling pathway assay as a simple and reliable means of identifying a suspected XIAP deficiency [34]. Moreover, given the influence of a *UNC13D* mutation on platelet granule exocytosis, flow cytometry detection of Munc13–4 protein expression in platelets can be used as an alternative approach for rapid FHL-3 screening [35].

With the wide application of whole exon sequencing (WES) and whole-genome sequencing (WGS), pHLH-related candidate genes are increasing in number; thus, additional new methods for cellular functional verification need to be explored and incorporated into the rapid pHLH diagnostic system/process.

## Conclusions

In summary, this large-scale retrospective study found the gene mutations, and the proportions of etiology are different between children, adolescents, and adult HLH patients in China. For the diagnosis of pHLH, the above immunological indicators had their different clinical prediction meanings and scope of applications. In clinical practice, appropriate cutoff values of immunological indicators may be essential for guiding prejudgment of pHLH.

## Supplementary information

**Additional file 1: Table S1.** Summary of patients with sequence variants in 12 pHLH related genes (*n*=128) [31, 33, 36–59].

## Data Availability

The datasets used during and/or analyzed during the current study are available from the corresponding author on request.

## Abbreviations

1000 g: 1000 Genomes Project
ACMG: American College of Medical Genetics and Genomics
ALFRED: ALlele FREquency Database
AUC: Area under the curve
CHS: Chediak–Higashi syndrome
CTL: Cytotoxic T lymphocytes
dbSNP: Single Nucleotide Polymorphism database
EBV: Epstein-Barr virus
ESP6500: NHLBI Exome Sequencing Project
ExAC: Exome Aggregation Consortium
FHL: Familial hemophagocytic lymphohistiocytosis
GS-2: Griscelli syndrome type 2
Hemi: Hemizygous
Het: Heterozygous
HGMD: Human Gene Mutation Database
Hom: Homozygous
HPS2: Hermansky-Pudlak syndrome type 2
LAMP-1: Lysosomal-associated membrane protein-1
LDH: Lactate dehydrogenase
NK: Natural killer
NPV: Negative predictive value
pHLH: primary hemophagocytic lymphohistiocytosis
PID: Primary immunodeficiency disorder
PPV: Positive predictive value
ROC: Receiver operating characteristic
SAP: SLAM associated protein
sHLH: secondary hemophagocytic lymphohistiocytosis
SIFT: Sorting Intolerant From Tolerant
WES: Whole exon sequencing
WGS: Whole genome sequencing
XIAP: X-linked inhibitor of apoptosis protein
