# Supplementary material for: Genotype characteristics and immunological indicator evaluation of 311 hemophagocytic lymphohistiocytosis cases in China
Source: Orphanet J Rare Dis. 2020 May 6;15:112. doi: 10.1186/s13023-020-01390-z (PMC7201972; doi:10.1186/s13023-020-01390-z)
Supplement: Supplementary file 1 — Additional file 1: Table S1. Summary of patients with sequence variants in 12 pHLH related genes (n=128) [31, 33, 36–59]. [file 13023_2020_1390_MOESM1_ESM.docx]

| **Supplementary Table S1** | | Summary of patients with sequence variants in 12 pHLH related genes (n=128) | | | | |  |  |  |  |  |  |
| --- | --- | --- | --- | --- | --- | --- | --- | --- | --- | --- | --- | --- |
| **Patient ID** | **Gender/Age at onset** | **Gene** | **Mutation** | **Genotype** | **ID** | **Pathogenic_Analysis** | **1000g2015aug_all** | **ExAC_ALL** | **esp6500siv2_all** | **SIFT_pred** | **Polyphen2_HVAR_pred** | **Publication** |
| 1 | M/6y | PRF1 | Exon3:c.C1066T(p.R356W) | Hom | rs746365230 | likely_pathogenic | - | 0.000008576 | - | D | B | Trizzino,et al^[36]^.2008 |
| 2 | M/19y | PRF1 | Exon3:c. C1349T(p.T450M) | Hom | rs189650890 | pathogenic | 0.000399361 | 0.00004957 | - | D | D | Ueda,et al^[37]^.2007 |
| 3 | M/27y | PRF1 | Exon2:c.65delC(p.P22RfsX29) | Compound | rs761651233 | likely_pathogenic | - | 0.0000108 | - | - | - | Tong,et al^[38]^.2011 |
|  |  |  | Exon2:c.G503A(p.S168N) | heterozygous | rs779399414 | likely_pathogenic | - | 0.00002473 | - | T | B | Lu,et al^[39]^.2009 |
| 4 | F/16y(Sibling) | PRF1 | Exon2:c.65delC(p.P22RfsX29) | Compound | rs761651233 | likely_pathogenic | - | 0.0000108 | - | - | - | Tong,et al^[38]^.2011 |
|  |  |  | Exon2:c.G503A(p.S168N) | heterozygous | rs779399414 | likely_pathogenic | - | 0.00002473 | - | T | B | Lu,et al^[39]^.2009 |
| 5 | F/16y(Sibling) | PRF1 | Exon2:c.65delC(p.P22RfsX29) | Compound | rs761651233 | likely_pathogenic | - | 0.0000108 | - | - | - | Tong,et al^[38]^.2011 |
|  |  |  | Exon2:c.G503A(p.S168N) | heterozygous | rs779399414 | likely_pathogenic | - | 0.00002473 | - | T | B | Lu,et al^[39]^.2009 |
| 6 | M/11y | PRF1 | Exon2:c.T172C(p.S58P) | Compound | - | uncertain | - | - | - | T | B | - |
|  |  |  | Exon3:c.1083_1094del(p.361_365del) | heterozygous | - | uncertain | - | - | - | - | - | - |
| 7 | F/9y | PRF1 | Exon2:c.G218A(p.C73Y) | Compound | rs759913385 | uncertain | - | 0.000008393 | - | D | D | - |
|  |  |  | Exon2:c.G394A(p.G132R) | heterozygous | rs201382038 | likely_pathogenic | - | 0.000008266 | - | D | P | Tong,et al^[38]^.2011 |
| 8 | F/17y | PRF1 | Exon2:c.A380G(p.N127S) | Compound | rs202091142 | uncertain | - | - | - | D | P | - |
|  |  |  | Exon3:c.853_855delAAG(p.K285del) | heterozygous | rs745902829 | likely_pathogenic | - | 0.00005766 | - | - | - | Göransdotter Ericson,et al^[40]^.2001 |
| 9 | F/20y | PRF1 | Exon2:c.G503A(p.S168N) | Compound | rs779399414 | likely_pathogenic | - | 0.00002473 | - | T | B | Lu,et al^[39]^.2009 |
|  |  |  | Exon3:c.C1349T(p.T450M) | heterozygous | rs189650890 | pathogenic | 0.000399361 | 0.00004957 | - | D | D | Ueda,et al^[37]^.2007 |
| 10 | F/1y | PRF1 | Exon3:c.C673T(p.R225W) | Compound | rs28933973 | likely_pathogenic | - | 0.00002481 | - | D | P | Stepp,et al^[41]^.1999 |
|  |  |  | Exon3:c.T1535G(p.L512R) | heterozygous | - | uncertain | - | - | - | D | P | - |
| 11 | M/2m | PRF1 | Exon3:c.853_855delAAG(p.K285del) | Compound | rs745902829 | likely_pathogenic | - | 0.00005766 | - | - | - | Göransdotter Ericson,et al^[40]^.2001 |
|  |  |  | Exon3:c.C1349T(p.T450M) | heterozygous | rs189650890 | pathogenic | 0.000399361 | 0.00004957 | - | D | D | Ueda,et al^[37]^.2007 |
| 12 | F/6y | PRF1 | Exon3:c.853_855delAAG(p.K285del) | Compound | rs745902829 | likely_pathogenic | - | 0.00005766 | - | - | - | Göransdotter Ericson,et al^[40]^.2001 |
|  |  |  | Exon3:c.C1349T(p.T450M) | heterozygous | rs189650890 | pathogenic | 0.000399361 | 0.00004957 | - | D | D | Ueda,et al^[37]^.2007 |
| 13 | M/3y | PRF1 | Exon3:c.G984A(p.W328X) | Compound | - | pathogenic | - | - | - | - | - | - |
|  |  |  | Exon3:c.C1349T(p.T450M) | heterozygous | rs189650890 | pathogenic | 0.000399361 | 0.00004957 | - | D | D | Ueda,et al^[37]^.2007 |
| 14 | F/2y | PRF1 | Exon3:c.1090_1091delCT(p.T364fsX93) | Compound | - | likely_pathogenic | - | - | - | - | - | Trizzino,et al^[36]^.2008 |
|  |  |  | Exon3:c.C1349T(p.T450M) | heterozygous | rs189650890 | pathogenic | 0.000399361 | 0.00004957 | - | D | D | Ueda,et al^[37]^.2007 |
| 15 | F/1y | PRF1 | Exon2:c.C10T(p.R4C) | Het | rs12161733 | likely_pathogenic | 0.00279553 | 0.0014 | 0.001 | D | B | My,et al^[42]^.2010 |
| 16 | M/10y9m | PRF1 | Exon2:c.65delC(p.P22RfsX29) | Het | rs761651233 | likely_pathogenic | - | 0.0000108 | - | - | - | Tong,et al^[38]^.2011 |
| 17 | M/14y | PRF1 | Exon2:c.G503A(p.S168N) | Het | rs779399414 | likely_pathogenic | - | 0.00002473 | - | T | B | Lu,et al^[39]^.2009 |
| 18 | M/13y | PRF1 | Exon2:c.G503A(p.S168N) | Het | rs779399414 | likely_pathogenic | - | 0.00002473 | - | T | B | Lu,et al^[39]^.2009 |
| 19 | M/3y | PRF1 | Exon2:c.G503A(p.S168N) | Het | rs779399414 | likely_pathogenic | - | 0.00002473 | - | T | B | Lu,et al^[39]^.2009 |
| 20 | M/24y | PRF1 | Exon3:c.C1005A(p.S335R) | Het | rs758844674 | uncertain | - | 0.000008295 | - | T | B | - |
| 21 | M/26y | PRF1 | Exon3:c.1419delC(p.T474QfsX5) | Het | - | pathogenic | - | - | - | - | - | - |
| 22 | F/27y | UNC13D | Exon27:c.G2588A(p.G863D) | Hom | rs140184929 | likely_pathogenic | 0.00139776 | 0.0004 | - | D | D | Tong,et al^[38]^.2011 |
| 23 | M/35y | UNC13D | Exon27:c.G2588A(p.G863D) | Hom | rs140184929 | likely_pathogenic | 0.00139776 | 0.0004 | - | D | D | Tong,et al^[38]^.2011 |
| 24 | M/52y | UNC13D | Exon27:c.G2588A(p.G863D) | Hom | rs140184929 | likely_pathogenic | 0.00139776 | 0.0004 | - | D | D | Tong,et al^[38]^.2011 |
| 25 | F/24y | UNC13D | Exon6:c.G407A(p.C136Y) | Compound | - | uncertain | - | - | - | D | D | - |
|  |  |  | Exon8:c.C640T(p.R214X) | heterozygous | rs769243366 | likely_pathogenic | - | 0.00000824 | - | - | - | Yamamoto,et al^[43]^.2004 |
| 26 | M/10m | UNC13D | Exon8: c.C640T(p.R214X) | Compound | rs769243366 | likely_pathogenic | - | 0.00000824 | - | - | - | Yamamoto,et al^[43]^.2004 |
|  |  |  | Exon30: c.2901_2902delGA(p.E967fs) | heterozygous | - | pathogenic | - | - | - | - | - | - |
| 27 | M/9y | UNC13D | Exon14:c.G1241T(p.R414L) | Compound | rs768171054 | likely_pathogenic | - | 0.000008282 | - | D | D | Santoro,et al^[44]^.2006 |
|  |  |  | Exon18-19: splicing c.1596+1G>C | heterozygous | - | pathogenic | - | - | - | - | - | Yamamoto,et al^[43]^.2004 |
| 28 | F/21y | UNC13D | Exon20:c.1832_1833insGTGCAGCGCGC(p.V612CfsX16) | Compound | - | pathogenic | - | - | - | - | - | - |
|  |  |  | Exon30-31:splicing c.2954+5G>A | heterozygous | - | pathogenic | - | - | - | - | - | Kim,et al^[45]^. 2010 |
| 29 | M/27y | UNC13D | Exon26-27:splicing c.2553+5C>G | Compound | rs201930023 | likely_pathogenic | 0.00299521 | 0.0008 | 0.0002 | - | - | Zhang,et al^[46]^.2011 |
|  |  |  | Exon31:c.C3134T(p.T1045M) | heterozygous | rs201146973 | uncertain | 0.000199681 | 0 | - | T | B | - |
| 30 | M/4y | UNC13D | Exon4: c.G305A(p.R102Q) | Het | rs778208597 | uncertain | - | 0.000008508 | - | T | B | - |
| 31 | M/6y | UNC13D | Exon8: c.C640T(p.R214X) | Het | rs769243366 | likely_pathogenic | - | 0.00000824 | - | - | - | Yamamoto,et al^[43]^.2004 |
| 32 | M/7y | UNC13D | Exon10:c.C760T(p.R254C) | Het | rs558726483 | uncertain | 0.000199681 | 0.0003 | - | D | P | - |
| 33 | M/11y | UNC13D | Exon13:c.C1120A(p.P374T) | Het | - | uncertain | - | - | - | T | P | Wang Y, et al^[47]^.2014 |
| 34 | F/6y | UNC13D | Exon14:c.A1228C(p.I410L) | Het | rs117221419 | uncertain | 0.00279553 | 0.001 | - | T | B | Tong,et al^[38]^.2011 |
| 35 | M/50y | UNC13D | Exon14:c.A1228C(p.I410L) | Het | rs117221419 | uncertain | 0.00279553 | 0.001 | - | T | B | Tong,et al^[38]^.2011 |
| 36 | F/14y | UNC13D | Exon14:c.A1228C(p.I410L) | Het | rs117221419 | uncertain | 0.00279553 | 0.001 | - | T | B | Tong,et al^[38]^.2011 |
| 37 | M/27y | UNC13D | Exon14:c.A1228C(p.I410L) | Het | rs117221419 | uncertain | 0.00279553 | 0.001 | - | T | B | Tong,et al^[38]^.2011 |
| 38 | F/9y3m | UNC13D | Exon14:c.A1228C(p.I410L) | Het | rs117221419 | uncertain | 0.00279553 | 0.001 | - | T | B | Tong,et al^[38]^.2011 |
| 39 | F/3y | UNC13D | Exon14:c.A1228C(p.I410L) | Het | rs117221419 | uncertain | 0.00279553 | 0.001 | - | T | B | Tong,et al^[38]^.2011 |
| 40 | F/24y | UNC13D | Exon14:c.A1228C(p.I410L) | Het | rs117221419 | uncertain | 0.00279553 | 0.001 | - | T | B | Tong,et al^[38]^.2011 |
| 41 | F/11y | UNC13D | Exon14:c.A1228C(p.I410L) | Het | rs117221419 | uncertain | 0.00279553 | 0.001 | - | T | B | Tong,et al^[38]^.2011 |
| 42 | M/2y | UNC13D | Exon21:c.1978_1979insATTACCG(p.V660fs) | Het | - | pathogenic | - | - | - | - | - | - |
| 43 | M/26y | UNC13D | Exon26-27:splicing c.2553+5C>G | Het | rs201930023 | likely_pathogenic | 0.00299521 | 0.0008 | 0.0002 | - | - | Zhang,et al^[46]^.2011 |
| 44 | F/29y | UNC13D | Exon27:c.G2588A(p.G863D) | Het | rs140184929 | likely_pathogenic | 0.00139776 | 0.0004 | - | D | D | Tong,et al^[38]^.2011 |
| 45 | M/5y | UNC13D | Exon27:c.G2588A(p.G863D) | Het | rs140184929 | likely_pathogenic | 0.00139776 | 0.0004 | - | D | D | Tong,et al^[38]^.2011 |
| 46 | M/1y4m | UNC13D | Exon29: c.C2782T(p.R928C) | Het | rs35037984 | uncertain | 0.0103834 | 0.0299 | 0.0159 | T | B | Santoro,et al^[44]^.2006 |
| 47 | M/48y | UNC13D | Exon31:c.C3067T(p.R1023C) | Het | rs140599939 | pathogenic | - | 0.0003 | - | T | B | - |
| 48 | M/3y | UNC13D | Exon31:c.C3067T(p.R1023C) | Het | rs140599939 | pathogenic | - | 0.0003 | - | T | B | - |
| 49 | F/29y | UNC13D | Exon32:c.3229_3235del(p.R1077fs) | Het | rs766652119 | likely_pathogenic | - | 0.0001 | - | - | - | - |
| 50 | M/14y | UNC13D | Exon32:c.3229_3235del(p.R1077fs) | Het | rs766652119 | likely_pathogenic | - | 0.0001 | - | - | - | - |
| 51 | F/1y7m | STX11 | Exon2:c.C121A(p.L41M) | Het | rs766869715 | uncertain | - | 0.00002488 | - | T | P | - |
| 52 | M/56y | STX11 | Exon2:c.C313A(p.R105S) | Het | - | uncertain | - | - | - | T | B | - |
| 53 | M/8y | STX11 | Exon2:c.C627A(p.S209R) | Het | - | uncertain | - | - | - | T | P | - |
| 54 | M/45 y | STX11 | Exon2:c.C646A(p.R216S) | Het | rs151047913 | uncertain | - | 0.00001675 | 0.000077 | D | B | - |
| 55 | F/31y | STX11 | Exon2:c.G799A(p.V267M) | Het | rs45574234 | uncertain | 0.00259585 | 0.0056 | 0.0065 | D | P | - |
| 56 | F/10m | STXBP2 | Exon14:c.G1214A(p.R405Q) | Hom | rs773360200 | likely_pathogenic | - | 0.00001673 | - | D | D | zur Stadt,et al^[48]^.2009 |
| 57 | M/17y | STXBP2 | Exon6:c.G359A(p.R120H) | Het | rs777213175 | uncertain | - | 0.000008243 | - | D | B | - |
| 58 | F/4y | STXBP2 | Exon6:c.390_392delGAA(p.K131del) | Het | - | uncertain | - | - | - | - | - | - |
| 59 | M/63y | STXBP2 | Exon7:c.C497T(p.T166M) | Het | rs181216956 | uncertain | 0.00259585 | 0.0002 | - | T | B | - |
| 60 | F/21y | STXBP2 | Exon11:c.C953T(p.T318M) | Het | rs201293382 | uncertain | 0.00119808 | 0.0004 | - | T | D | - |
| 61 | M/16y | STXBP2 | Exon13:c.C1034T(p.T345M) | Het | rs117761837 | uncertain | 0.00459265 | 0.0109 | 0.0118 | D | D | Al Hawas,et al^[49]^.2012 |
| 62 | M/3y9m | STXBP2 | Exon14:c.G1204A(p.D402N) | Het | rs114628602 | uncertain | 0.000199681 | 0.0002 | 0.0002 | T | P | - |
| 63 | M/1y | STXBP2 | Exon16:c.C1375T(p.R459W) | Het | rs142105943 | uncertain | 0.00159744 | 0.006 | 0.0003 | D | P | - |
| 64 | F/8y | STXBP2 | Exon18:c.A1663G(p.R555G) | Het | rs61736586 | uncertain | 0.0121805 | 0.0043 | 0.0089 | D | B | Mukda,et al^[50]^.2017 |
| 65 | F/34y | RAB27A | Exon5:c.C244T(p.R82C) | Hom | rs753966933 | likely_pathogenic | - | 0.000008303 | - | D | D | Netter,et al^[51]^.2016 |
| 66 | M/7y | RAB27A | Exon5:c.C244T(p.R82C) | Compound | rs753966933 | likely_pathogenic | - | 0.000008303 | - | D | D | Netter,et al^[51]^.2016 |
|  |  |  | Exon6:c.377delC(p.P126QfsX3) | heterozygous | - | Pathogenic | - | - | - | - | - | - |
| 67 | F/29y | RAB27A | Exon3:c.G11T(p.G4V) | Het | rs539575657 | uncertain | 0.000199681 | 0.000074 | - | T | B | - |
| 68 | M/60y | LYST | Exon5:c.A368G(p.H123R) | Het | rs3768067 | likely_pathogenic | 0.00219649 | 0.0007 | - | T | B | Mukda,et al^[50]^.2017 |
| 69 | F/2y | LYST | Exon5:c.A368G(p.H123R) | Het | rs3768067 | likely_pathogenic | 0.00219649 | 0.0007 | - | T | B | Mukda,et al^[50]^.2017 |
| 70 | F/37y | LYST | Exon5:c.A368G(p.H123R) | Het | rs3768067 | likely_pathogenic | 0.00219649 | 0.0007 | - | T | B | Mukda,et al^[50]^.2017 |
| 71 | F/7y | LYST | Exon5:c.T2255A(p.L752Q) | Het | rs545240869 | uncertain | - | 0.0000165 | - | D | D | - |
| 72 | F/23y | LYST | Exon13:c.A4573G(p.I1525V) | Het | rs190647059 | uncertain | 0.000199681 | 0.000008276 | - | T | B | - |
| 73 | F/54y | LYST | Exon24:c.A6986G(p.K2329R) | Het | rs532692084 | uncertain | 0.000199681 | 0.000008255 | - | T | B | - |
| 74 | F/21y | LYST | Exon25:c.C7159G(p.R2387G) | Het | - | uncertain | - | - | - | T | B | - |
| 75 | F/25y | LYST | Exon27:c.T7586A(p.M2529K) | Het | - | uncertain | - | - | - | D | B | - |
| 76 | F/22y | LYST | Exon30:c.A7994G(p.D2665G) | Het | rs562418362 | uncertain | 0.000998403 | 0.00008315 | - | T | B | - |
| 77 | F/58y | LYST | Exon32:c.A8368C(p.K2790Q) | Het | rs138506576 | uncertain | 0.00199681 | 0.0008 | - | D | B | - |
| 78 | F/38y | LYST | Exon32:c.A8368C(p.K2790Q) | Het | rs138506576 | uncertain | 0.00199681 | 0.0008 | - | D | B | - |
| 79 | F/30y | LYST | Exon32:c.A8368C(p.K2790Q) | Het | rs138506576 | uncertain | 0.00199681 | 0.0008 | - | D | B | - |
| 80 | F/44y | LYST | Exon32:c.A8368C(p.K2790Q) | Het | rs138506576 | uncertain | 0.00199681 | 0.0008 | - | D | B | - |
| 81 | M/29y | LYST | Exon32:c.A8368C(p.K2790Q) | Het | rs138506576 | uncertain | 0.00199681 | 0.0008 | - | D | B | - |
| 82 | M/38y | LYST | Exon32:c.A8368C(p.K2790Q) | Het | rs138506576 | uncertain | 0.00199681 | 0.0008 | - | D | B | - |
| 83 | M/15y | LYST | Exon32:c.A8368C(p.K2790Q) | Het | rs138506576 | uncertain | 0.00199681 | 0.0008 | - | D | B | - |
| 84 | F/14y | LYST | Exon32:c.C8514A(p.D2838E) | Het | rs376817988 | uncertain | - | 0.00002472 | - | T | B | - |
| 85 | M/5y | LYST | Exon34:c.G8624A(p.R2875H) | Het | rs200353560 | uncertain | 0.000399361 | 0.0002 | 0.000077 | D | D | - |
| 86 | M/36y | LYST | Exon46:c.G10526A(p.R3509Q) | Het | rs138936105 | uncertain | 0.00139776 | 0.0003 | 0.0009 | T | B | - |
| 87 | M/16y | LYST | Exon49:c.T10833A(p.H3611Q) | Het | - | uncertain | - | - | - | T | D | - |
| 88 | F/11y | AP3B1 | Exon9:c.G1031A(p.R344H) | Het | rs765852823 | uncertain | - | 0.0000165 | - | T | P | - |
| 89 | F/26y | AP3B1 | Exon21:c.A2417G(p.K806R) | Het | - | uncertain | - | - | - | T | B | - |
| 90 | M/2y | AP3B1 | Exon27:c.C3197T(p.S1066F) | Het | rs764100439 | uncertain | - | 0.0000659 | - | D | B | - |
| 91 | M/11m | SH2D1A | Exon2:c.C163T(p.R55X) | Hemi | rs111033623 | pathogenic | - | - | - | - | - | Coffey,et al^[52]^.1998 |
| 92 | M/1y8m | SH2D1A | Exon2:c.C163T(p.R55X) | Hemi | rs111033623 | pathogenic | - | - | - | - | - | Coffey,et al^[52]^.1998 |
| 93 | F/20y | SH2D1A | Exon1:c.G7T(p.A3S) | Het | rs148554414 | likely_pathogenic | 0.000529801 | 0.0002 | 0.000095 | T | B | Zhao,et al^[53]^.2011 |
| 94 | F/36y | SH2D1A | Exon3:c.A265G(p.K89E) | Het | - | uncertain | - | - | - | D | B | - |
| 95 | F/1y | SH2D1A | Exon3:c.G304A(p.V102I) | Het | rs200689267 | uncertain | 0.000264901 | 0.00002281 | - | T | D | - |
| 96 | M/1y | BIRC4 | Exon2:c.A224G(p.Y75C) | Hemi | - | uncertain | - | - | - | T | P | - |
|  |  |  | Exon2:c.615delA(p.G205GfsX3) |  | - | pathogenic | - | - | - | - | - | - |
| 97 | M/7y | BIRC4 | Exon2:c.G592A(p.V198M) | Hemi | - | uncertain | - | - | - | D | D | - |
| 98 | M/6m | BIRC4 | Exon2:c.A659T(p.H220L) | Hemi | - | uncertain | - | - | - | D | D | - |
| 99 | M/4y | BIRC4 | Exon3:c.878_977del(p.G293fs) | Hemi | - | pathogenic | - | - | - | - | - | - |
| 100 | M/70y | ITK | Exon13:c.G1343A(p.R448H) | Het | rs144735141 | likely_pathogenic | - | 0.0002 | - | D | P | Kanchi,et al^[54]^.2014 |
| 101 | M/25y | ITK | Exon16:c.C1741T(p.R581W) | Het | rs34482255 | likely_pathogenic | 0.000998403 | 0.0008 | 0.0003 | D | D | Kanchi,et al^[54]^.2014 |
| 102 | F/18y | ITK | Exon16:c.C1741T(p.R581W) | Het | rs34482255 | likely_pathogenic | 0.000998403 | 0.0008 | 0.0003 | D | D | Kanchi,et al^[54]^.2014 |
| 103 | M/7m | ITK | Exon16:c.C1741T(p.R581W) | Het | rs34482255 | likely_pathogenic | 0.000998403 | 0.0008 | 0.0003 | D | D | Kanchi,et al^[54]^.2014 |
| 104 | M/1y | ITK | Exon16:c.C1741T(p.R581W) | Het | rs34482255 | likely_pathogenic | 0.000998403 | 0.0008 | 0.0003 | D | D | Kanchi,et al^[54]^.2014 |
| 105 | M/1y | ITK | Exon16:c.C1741T(p.R581W) | Het | rs34482255 | likely_pathogenic | 0.000998403 | 0.0008 | 0.0003 | D | D | Kanchi,et al^[54]^.2014 |
| 106 | F/27y | ITK | Exon16:c.G1759A(p.V587I) | Het | rs56005928 | uncertain | 0.00179712 | 0.0034 | 0.0019 | T | B | - |
| 107 | F/5y | MAGT1 | Exon1:c.G192C(p.K64N) | Het | - | uncertain | - | - | - | D | B | - |
| 108 | M/36y | PRF1/UNC13D | **PRF1:**Exon2:c.G133A(G45R); | Compound | rs578092914 | likely_pathogenic | 0.000199681 | - | - | D | D | Kogawa,et al^[55]^.2002 |
|  |  |  | Exon3:c.C1228T(R410W) | Heterozygous/ | rs139322149 | likely_pathogenic | 0.000199681 | 0.00005111 | 0.000077 | D | D | Ueda,et al^[37]^.2007 |
|  |  |  | **UNC13D**:Exon14:c.G1280A(R427Q) | Het | rs200661413 | uncertain | 0.000199681 | 0.00008298 | 0.000077 | D | D | - |
| 109 | M/3y | PRF1/UNC13D | **PRF1**:Exon3:c.C1349T(p.T450M) | Het | rs189650890 | pathogenic | 0.000399361 | 0.00004957 | - | D | D | Ueda,et al^[37]^.2007 |
|  |  |  | **UNC13D:**Exon14:c.A1228C(p.I410L) |  | rs117221419 | uncertain | 0.00279553 | 0.001 | - | T | B | Tong,et al^[38]^.2011 |
| 110 | F/8y | PRF1/STXBP2 | **PRF1**:Exon2:c.C10T(p.R4C); | Het(Monoallelic)/ | rs12161733 | likely_pathogenic | 0.00279553 | 0.0014 | 0.001 | D | B | My,et al^[42]^.2010 |
|  |  |  | Exon2:c.G98A(p.R33H) | Het | rs531407289 | likely_pathogenic | 0.000199681 | 0.0001 | - | D | B | Zhang,et al^[46]^.2011 |
|  |  |  | **STXBP2:**Exon4:c.C190T(p.R64W) |  | rs764937841 | uncertain | - | 0.00003395 | - | D | D | - |
| 111 | F/30y | PRF1/STXBP2 | **PRF1:**Exon2: c.G503A(p.S168N) | Het/ | rs779399414 | likely_pathogenic | - | 0.00002473 | - | T | B | Lu,et al^[39]^.2009 |
|  |  |  | **STXBP2:**Exon4:c.A184G(p.N62D); | Het(Monoallelic) | rs368283130 | uncertain | - | 0.00005105 | - | T | P | - |
|  |  |  | Exon7:c.C497T(p.T166M) |  | rs181216956 | uncertain | 0.00259585 | 0.0002 | - | T | B | - |
| 112 | F/17y | PRF1/LYST | **PRF1**:Exon2:c.C46T(p.P16S); | Compound | - | uncertain | - | - | - | T | P | - |
|  |  |  | Exon3:c.C1066T(p.R356W) | Heterozygous/ | rs746365230 | likely_pathogenic | - | 0.000008576 | - | D | B | Trizzino,et al^[36]^.2008 |
|  |  |  | **LYST:**Exon15:c.T4972C(p.F1658L); | Het(Monoallelic) | rs771710044 | uncertain | - | 0.000008267 | - | T | B | - |
|  |  |  | Exon45:c.A10285G(p.I3429V) |  | rs769142815 | uncertain | - | 0.000008237 | - | T | B | - |
| 113 | F/32y | UNC13D/STX11 | **UNC13D:**Exon14:c.A1228C(p.I410L) | Het | rs117221419 | uncertain | 0.00279553 | 0.001 | - | T | B | Tong,et al^[38]^.2011 |
|  |  |  | **STX11:**Exon2:c.A326G(p.E109G) |  | - | uncertain | - | - | - | T | B | - |
| 114 | F/1y | UNC13D/STXBP2 | **UNC13D:**Exon14:c.A1228C(p.I410L) | Het | rs117221419 | uncertain | 0.00279553 | 0.001 | - | T | B | Tong,et al^[38]^.2011 |
|  |  |  | **STXBP2**:Exon18:c.A1663G(p.R555G) |  | rs61736586 | uncertain | 0.0121805 | 0.0043 | 0.0089 | D | B | Mukda,et al^[50]^.2017 |
| 115 | M/8y | UNC13D/LYST | **UNC13D:**Exon12-13:splicing c.1055+1G>A; | Compound | rs754205110 | likely_pathogenic | - | 0.000008329 | - | - | - | Sieni,et al^[56]^.2011 |
|  |  |  | Exon31:c.G2986A(p.G996R) | Heterozygous/ | - | uncertain | - | - | - | D | D | - |
|  |  |  | **LYST:**Exon51:c.A11159G(p.N3720S) | Het | - | uncertain | - | - | - | D | D | - |
| 116 | M/2y | UNC13D/LYST | **UNC13D:**Exon14:c.G1189A(p.A397T) | Het | rs769602840 | uncertain | - | 0.0000827 | - | T | B | - |
|  |  |  | **LYST:**Exon34:c.G8624A(p.R2875H) |  | rs200353560 | uncertain | 0.000399361 | 0.0002 | 0.000077 | D | D | - |
| 117 | F/19y | UNC13D/LYST | **UNC13D**:Exon14:c.A1228C(p.I410L) | Het | rs117221419 | uncertain | 0.00279553 | 0.001 | - | T | B | Tong,et al^[38]^.2011 |
|  |  |  | **LYST:**Exon21:c.G6114C(p.L2038F) |  | - | uncertain | - | - | - | D | D | - |
| 118 | M/27y | UNC13D/LYST | **UNC13D**:Exon14:c.G1232A(p.R411Q) | Het | rs200109035 | likely_pathogenic | 0.000798722 | 0.0006 | 0.000077 | T | B | Gao,et al^[57]^.2015 |
|  |  |  | **LYST:**Exon5:c.A368G(p.H123R) |  | rs3768067 | likely_pathogenic | 0.00219649 | 0.0007 | - | T | B | Mukda,et al^[50]^.2017 |
| 119 | F/2y | UNC13D/LYST | **UNC13D:**Exon18-19:splicing:c.1597-2A>G | Het | - | uncertain | - | - | - | - | - | - |
|  |  |  | **LYST:**Exon32:c.A8368C(p.K2790Q) |  | rs138506576 | uncertain | 0.00199681 | 0.0008 | - | D | B | - |
| 120 | F/22y | UNC13D/LYST | **UNC13D:**Exon28-29:splicing:c.2709+1G>A | Het | - | pathogenic | - | - | - | - | - | Zhizhuo,et al^[58]^.2012 |
|  |  |  | **LYST:**Exon13:c.T4578A(p.N1526K) |  | rs117609949 | uncertain | 0.000399361 | 0.0002 | - | T | B | - |
| 121 | F/30y | UNC13D/LYST | **UNC13D**:Exon30:c.C2896T(p.R966W); | Het | rs118049905 | likely_pathogenic | 0.00179712 | 0.0096 | 0.0051 | D | D | Zhang,et al^[31]^.2014 |
|  |  |  | **LYST:**Exon13:c.T4578A(p.N1526K) |  | rs117609949 | uncertain | 0.000399361 | 0.0002 | - | T | B | - |
| 122 | M/31y | UNC13D/SH2D1A | **UNC13D**:Exon27:c.G2588A(p.G863D) | Het/Hemi | rs140184929 | likely_pathogenic | 0.00139776 | 0.0004 | - | D | D | Tong,et al^[38]^.2011 |
|  |  |  | **SH2D1A:**Exon1:c.T32G(p.I11S) |  | - | uncertain | - | - | - | D | D | Zhang,et al^[59]^.2016 |
| 123 | F/32y | UNC13D/LYST/BIRC4 | **UNC13D:**Exon8:c.G680A(p.R227H) | Het | rs751394792 | uncertain | - | 0.00006594 | - | T | P | - |
|  |  |  | **LYST:**Exon32:c.A8368C(p.K2790Q) |  | rs138506576 | uncertain | 0.00199681 | 0.0008 | - | D | B | - |
|  |  |  | **BIRC4:**Exon3:c.C962G(p.A321G) |  | rs182340753 | likely_pathogenic | 0.000794702 | 0.0003 | - | D | D | Gifford,et al^[33]^.2014 |
| 124 | M/1y | UNC13D/LYST/BIRC4 | **UNC13D:**Exon31:c.C3067T(p.R1023C) | Het/Hemi | rs140599939 | pathogenic | - | 0.0003 | - | T | B | - |
|  |  |  | **LYST:**Exon30:c.A7994G(p.D2665G) |  | rs562418362 | uncertain | 0.000998403 | 0.00008315 | - | T | B | - |
|  |  |  | **BIRC4:**c.1038_1041delTTCA(p.S347Lfs) |  | - | pathogenic | - | - | - | - | - | - |
| 125 | F/5m | UNC13D/ITK | **UNC13D:** Exon14:c.A1228C(p.I410L); | Compound | rs117221419 | uncertain | 0.00279553 | 0.001 | - | T | B | Tong,et al^[38]^.2011 |
|  |  |  | Exon31:c.C3067T(p.R1023C) | Heterozygous/Het | rs140599939 | pathogenic | - | 0.0003 | - | T | B | - |
|  |  |  | **ITK:**Exon16:c.C1741T(p.R581W) |  | rs34482255 | likely_pathogenic | 0.000998403 | 0.0008 | 0.0003 | D | D | Kanchi,et al^[54]^.2014 |
| 126 | F/29y | STXBP2/LYST | **STXBP2:**Exon11:c.C953T(p.T318M) | Het | rs201293382 | uncertain | 0.00119808 | 0.0004 | - | T | D | - |
|  |  |  | **LYST:**Exon32:c.A8368C(p.K2790Q) |  | rs138506576 | uncertain | 0.00199681 | 0.0008 | - | D | B | - |
| 127 | M/3y | STXBP2/LYST/BIRC4 | **STXBP2:**Exon7:c.C497T(p.T166M) | Het/Hemi | rs181216956 | uncertain | 0.00259585 | 0.0002 | - | T | B | - |
|  |  |  | **LYST**:Exon32:c.A8368C(p.K2790Q) |  | rs138506576 | uncertain | 0.00199681 | 0.0008 | - | D | B | - |
|  |  |  | **BIRC4:** Exon2:c.G592C(p.V198L) |  | - | uncertain | - | - | - | D | D | - |
| 128 | M/9y | STXBP2/MAGT1 | **STXBP2:**Exon18:c.A1663G(p.R555G) | Het/Hemi | rs61736586 | uncertain | 0.0121805 | 0.0043 | 0.0089 | D | B | Mukda,et al^[50]^.2017 |
|  |  |  | **MAGT1:** Exon5:c.C755A(p.A252D); |  | - | uncertain | - | - | - | D | D | - |
|  |  |  | Exon5:c.759delT(p.F253fsX10) |  | - | pathogenic | - | - | - | - | - | - |
|  |  |  |  |  |  |  |  |  |  |  |  |  |
| **Notes to table contents：** | | |  |  |  |  |  |  |  |  |  |  |
| M:male F:female y:year m:month Hom:homozygous Het:heterozygous Hemi:hemizygous 1000g:1000 Genomes Project ExAC:Exome Aggregation Consortium esp6500:NHLBI Exome Sequencing Project  SIFT_pred: D(amaging); T(olerated) Polyphen2_HVAR_pred: D (probably damaging), P (possibly damaging) and B (benign) | | | | | | | | | | | | |
